# Wearable sensors and VOC monitoring: data fusion and machine learning for non-invasive disease detection

**DOI:** 10.1088/1752-7163/aeb685

**Published:** 2026-11-04

**Authors:** Reid Honeycutt, Flore M Hervé, Patrick Gibson, Eva Borras, Dante E Rojas, Yevgeniy Belous, Stephanie Fung, Mitchell M McCartney, Nicholas J Kenyon, Cristina E Davis

**Affiliations:** 1 Mechanical and Aerospace Engineering, University of California, Davis, CA, United States of America; 2 UC Davis Lung Center, Davis, CA, United States of America; 3 VA Northern California Health Care System, Mather, CA, United States of America; 4 Division of Pulmonary, Critical Care and Sleep Medicine, University of California Davis, Sacramento, CA 95816, United States of America

**Keywords:** breath analysis, volatile organic compounds (VOCs), wearable sensors, data fusion, asthma

## Abstract

Emerging technologies that detect volatile organic compounds (VOCs) emanating from the body enable rapid and non-invasive detection of diseases. Respiratory illnesses, in particular, can be identified through measurement of VOCs in exhaled breath. However, the low specificity of some methods leaves room for improvement. By combining information from multiple data streams, we can potentially improve on the results obtained from using each data stream individually. Here we evaluate a novel data fusion approach combining a wearable vitals monitor and exhaled breath VOCs for the classification of asthma patients. The wearable vitals monitor or ‘e-glove’ measures key information such as skin temperature, trans-epidermal water loss, heart rate, and galvanic skin response, while the breath VOC data (measured by gold standard gas chromatography-mass spectrometry) provide insight into the biochemical and metabolic state of the body. Data fusion is a process used in many applications for combining information from disparate sources. We leverage data fusion in this case to achieve superior classification accuracy using the integrated data streams. The low-level data fusion achieved a higher ROC AUC score (0.88) in asthma classification than the breath VOC-only approach (0.72, *p* < 0.001). These results are promising for future data analysis workflows for non-invasive diagnostic and health monitoring.

## 1. Introduction

Breath biomarker analysis is a developing technology for the diagnosis and monitoring of diseases. Exhaled breath vapor (EBV) is a major fraction of the breath, which contains hundreds of volatile organic compounds (VOCs). Compounds in the breath may be inhaled from the environment, absorbed through the skin, or produced endogenously by the body’s own cells or by microbes [1]. Endogenous VOCs provide insight into the metabolic processes in the body and can indicate disease or injury [2]. Clinical screening for illnesses such as SARS-CoV-2 and influenza have been investigated using breath metabolomics [3, 4].

As an inflammatory airway disease, asthma lends itself to the study of breath diagnostics. We have previously performed a prospective clinical study in a cohort of adolescent asthma subjects to identify altered metabolite profiles in exhaled breath condensate, another fraction of the breath [5], and other investigators have also shown altered oxidative stress markers in the breath of asthma patients [6–8]. Asthma is a chronic condition characterized by inflammation of the bronchioles in the lower airway and is one of the most common diseases in the world. Additionally, asthma is frequently under-treated and under-diagnosed, especially in developing countries [9]. There are many methods currently used to help diagnose asthma, including patient history, blood or sputum eosinophil count, fraction exhaled nitric oxide measurement, bronchodilator reversibility tests, and spirometry [10]. Generally, diagnosis requires both a compatible patient history and either demonstration of variability of limitations in expiratory flowrate or evidence of high level of exhaled nitric oxide (FeNO > 50 ppb, ERS guidelines [11]) indicative of airway inflammation. However, there are different asthma phenotypes, resulting from different mechanisms of disease. This means there is no single gold standard criterion for asthma diagnosis. Indeed, in older patients, it can be difficult for clinicians to differentiate asthma from other respiratory disorders, such as COPD [12]. Therefore, it is desirable to develop more accurate ways to monitor these diseases.

There is substantial interest in continuous monitoring of asthma severity and predicting when symptoms may worsen. Current approaches to asthma monitoring include daily symptom questionnaires, smartphone apps that account for factors such as weather and pollen, and vital monitoring approaches [13]. Vitals can be monitored through contactless devices, such as camera and radar-based sensors [14], smart textiles that embed sensors directly into fabric and clothing items [15], and skin-contact sensors that operate similarly to clinical devices [16]. Commonly collected vitals for monitoring asthma include body temperature, blood pressure, blood oxygen saturation, heart rate, breathing rate, and airway sound [13].

In parallel to our breath metabolomics work, we present a wearable e-glove vitals monitoring system which can simultaneously measure skin temperature, trans-epidermal water loss (TEWL), heart rate, and galvanic skin response (GSR) (figure 1). The goal of this device was to develop a portable means to simultaneously measure multiple vital parameters, to improve collection of these data in settings that may not have access to clinical gold standard tools. In this work, we compare the inclusion of vitals data streams with exhaled VOC data to investigate whether data fusion approaches can improve model accuracy to screen for asthma conditions.

**Figure 1.**
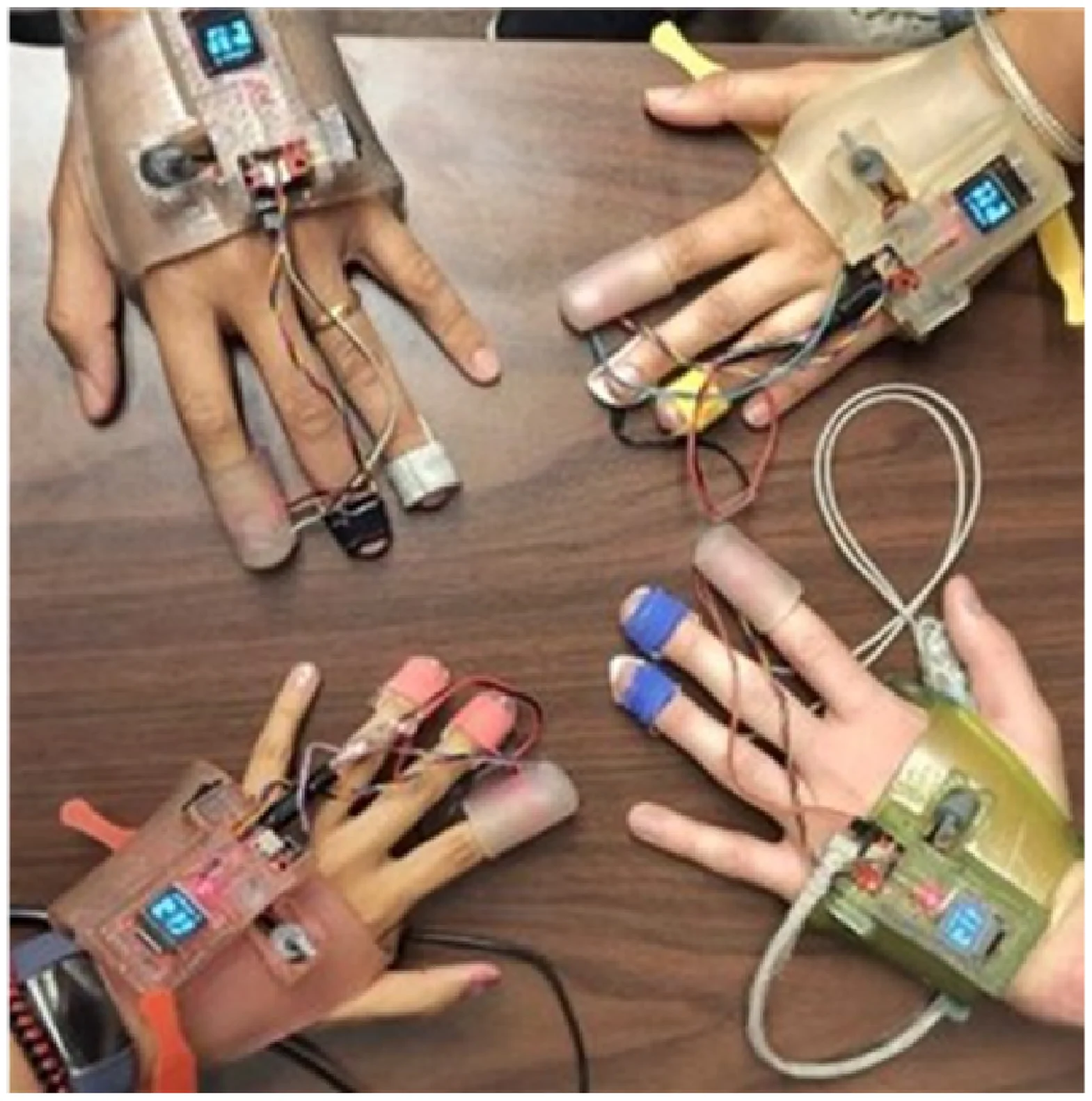
Multiple e-gloves being worn by patients with various hand sizes.

These vital signals provide insight into the internal state of the body and potentially indicate physiological stress. Various wearable vitals monitoring devices have been trialed for monitoring asthma severity in patients. Exacerbations in symptoms may take place over the course of days or even in the space of a few hours [13]. Worsening symptoms can include coughing, wheezing, difficulty breathing, and tightness in the chest [6]. Each of the vital signals chosen has an association with the presence or severity of asthma symptoms. TEWL can be a measure of physiological stress, as it is modulated by eccrine gland activity. However, some emerging evidence has also associated abnormalities in TEWL levels with atopic conditions such as allergies and asthma, possibly as a result of dysfunctional epithelial barriers [17].

Heart rate is an important metric in monitoring the severity of asthma in sufferers, particularly in detecting an acute asthmatic episode. When experiencing symptoms, heart rate typically increases dramatically [18]. Given that increased heart rate can result from worsening symptoms, heart rate was selected as a metric for distinguishing asthmatic patients from healthy controls. Although body temperature does not appear to be directly related to worsening asthma symptoms, it is often monitored as a standard vital metric [13].

GSR, or electrodermal activity (EDA), has been shown to be affected by many health conditions, including chronic respiratory conditions such as COPD [19]. EDA is modulated by the sweat glands which are innervated by the sympathetic nervous system. Measuring changes in EDA provides insight into the activity of the sudomotor nerves that stimulate sweat production and the arousal of the autonomic nervous system [20]. Data fusion techniques vary in complexity. Low-level data fusion includes techniques such as concatenation of data vectors from different instruments and computation of the outer product of these vectors [21]. Data fusion can also be performed at the feature level, in which engineered or extracted features from disparate sources are combined [22]. In decision level fusion, the outputs of classifiers trained from different data are fused to obtain the final class prediction. While both vitals monitoring and breath biomarker analysis are promising candidates for developing a health monitoring model for asthma, by fusing these data we test if it is possible to obtain superior classification accuracy.

In this work, we leverage both low-level and mid-level data fusion approaches to combine patient vital statistics and breath metabolite analysis to produce an improved health monitoring model. We use asthma as a good case study for this approach. We hypothesize that a fusion data approach will have greater power to distinguish between subjects with asthma and non-asthmatic controls compared to either model alone.

## 2. Materials and methods

### 2.1. Patient recruitment

Subjects with and without asthma were recruited (*n* = 39, 19 control, 20 asthmatic) from specialty asthma clinics at the University of California, Davis Health System, and from volunteers at the UC Davis campus. All patients in the asthma cohort had been previously diagnosed by a physician. All subjects provided informed consent for the IRB-approved study (UC Davis IRB #1859852). All patients were adults over 18. 17 of the 20 subjects in the asthma cohort had other comorbid diseases including chronic lung disease, chronic obstructive pulmonary disease, sleep apnea, hypertension, diabetes, osteoarthritis, rheumatoid arthritis, cancer, and others. All but one subject in the non-asthma control group were free of other comorbid diagnoses, except for one control patient with a sleep apnea diagnosis. The average age of asthma patients was 50.6 ± 20.5 years, while the average age of the control group was 32.1 ± 12.4 years. In the control group there were 7 male and 12 female patients, while in the asthma group there were 5 male and 15 female patients.

### 2.2. E-Glove operating principle

Figure 2 shows the overall structure of the wearable vitals monitor, the e-glove. The flexible main body of the 3D resin printed glove (Formlabs, elastic 50A resin) was designed to house 4 different vitals sensors, measuring the skin temperature, TEWL, heart rate, and GSR. A commercial Arduino-based microcontroller for controlling glove functions and processing, storing, and communicating sensor data is also housed within the glove. Additional design considerations were portability and comfort of wearing the glove.

**Figure 2.**
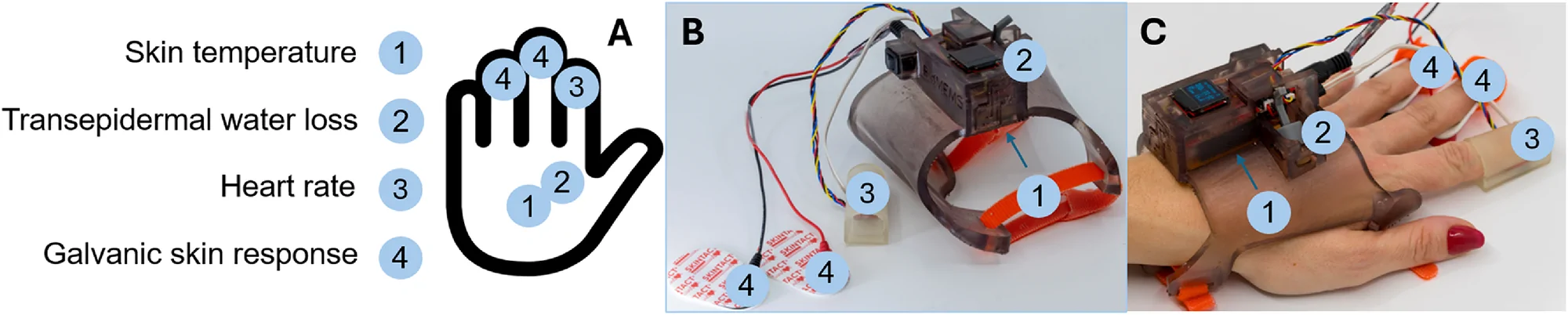
Sensor placement on the e-glove. (A) Positions of vital sensors on the hand. (B) Side view of the e-glove. The flexible body of the glove can be adjusted to fit different patients. All vital sensors are mounted directly on the glove. (C) The e-glove being worn.

#### 2.2.1. E-glove hardware

A programmable microcontroller (THING PLUS ESP32 WROOM USB-C, WRL-20168, Sparkfun Electronics) was used to control and gather data from the sensors through I^2^C communication. The microcontroller also offers a built-in micro-SD port and Bluetooth low energy (BLE) connectivity. The data was collected at 20 Hz sampling rate, stored in a 16 GB microSD card installed in the microcontroller (SanDisk Corporation, Milpitas, CA, USA), and simultaneously transmitted via BLE communication to a computer for redundancy in data collection. In addition, an OLED screen (Sparkfun) was mounted on the e-glove for real-time data monitoring to ensure proper installation of the glove and verify data collection during the study. The board was powered by a 3.7 V, 650 mAh lithium-ion battery (YDl, Dongguan Yongdeli Electronic Co.) to enable portability. Arduino IDE code libraries were provided by the manufacturers of the sensors and used in the script for running the e-glove. For the integration of the electronics and sensors, custom printed circuit boards (PCBs) (supplementary figure S1) were designed in KiCad and assembled and tested in-house.

### 2.3. E-glove vital sensors

#### 2.3.1. Temperature sensor

A digital temperature sensor (985-AS6221-AWLT-S, ams-OSRAM USA INC.) was used to measure the skin temperature. It was placed directly on the PCB for direct contact with the skin on the back of the hand. A temperature accuracy of ±0.4 °C was validated through measurements of two areas on the body, the forehead and forearm, as taken with an IR forehead thermometer (Famidoc FDIR-V22, Thermobio) as the gold standard for comparison and validation. Two different measurement modes for the IR thermometer were used, one with a bias built in for accurately measuring skin temperature, and one without bias. The temperature sensor was compared to both modes to calibrate it for measuring skin temperature (supplementary figure S2).

#### 2.3.2. TEWL sensor

The TEWL sensor uses the closed-chamber method of TEWL measurement [23]. For this purpose, a cylindrical chamber (D:10 mm, H:10 mm) was 3D-printed in latex-free resin. On the inside of the chamber, at the top of the cylinder, the humidity and temperature sensor (SHT45, Sensirion) was placed. The change in relative humidity (RH%) inside the chamber was measured to calculate the TEWL. When the chamber is in contact with the skin, water evaporated from the skin collects, and the sensor measures the increase in relative humidity over time. During skin contact, the humidity level rises linearly for a specific range of time. The rate at which the humidity rises (Δ%/Δ*t*) in the linear phase is calculated and is proportional to the TEWL (supplementary figure S3).

To characterize the TEWL measurement performance of the proposed device, a calibration based on the wet-cup method was performed. The wet-cup method is widely accepted as one of the general calibration methods for the TEWL measurement, providing a quantitative water loss in units of g m^−2^ h^−1^ [24]. The artificial skin was composed of a semi-permeable membrane (OpSite Flexigrid, Smith and Nephew, England) covering a petri dish with water (D: 89 mm). The reference TEWL is generated through the membrane and is controlled by changes in water temperature. The water temperature was changed with a hot plate. Supplementary figure S3A shows the reference TEWL values obtained by the artificial TEWL skin for 3 different conditions, with the water temperature ranging from 20 °C–40 °C. The developed TEWL sensor was then compared for validation against the gold-standard sensor (Delfin) on the back of the hand (supplementary figure S3D).

#### 2.3.3. Heart rate sensor

A photoplethysmography-based sensor (SEN-15219, SparkFun Electronics) was selected for reading the heart rate. To provide consistent pressure for accurate measurement, a housing was created to hold the sensor against the fingertip. The housing was designed to hold the sensor board in the bottom and the finger at the top (supplementary figure S4). Five sizes of the fingertip housing were produced to accommodate different hand sizes. The sensor gives the heart rate in beats per minute and the detection status (no detection, over pressure, detection). The sensor was evaluated during 3 different activities: at rest, during flexion-extension, and during a sprint in triplicate with 3 different patients. The results obtained were compared against an AccuMed pulse oximeter for validation of the sensor.

#### 2.3.4. GSR sensor

A commercial sensor was not used for measurement of skin conductivity. A voltage divider was built directly into the PCB (supplementary figure S1), composed of a resistor, operational amplifier, and two downstream electrodes in contact with fingers. Two gel electrodes (Skintact, MFI Medical) were placed on the tips of the index and middle fingers and secured with a strap. The skin resistance (${R_{{\mathrm{skin}}}}$) is calculated with equation (1) and the inverse of resistance, skin conductivity (${G_{{\mathrm{skin}}}}$), measured in microsiemens (*μ*S), is determined with equation (2),

\begin{equation*}\begin{array}{*{20}{c}} {{R_{{\mathrm{skin}}}} = \frac{{{ }2{\ast}{V_{\mathrm{s}}}{\ast}{R_6}}}{{{\mathrm{GS}}{{\mathrm{R}}_{{\mathrm{OUT}}}}}} - {R_6}} \end{array}\end{equation*}

\begin{equation*}\begin{array}{*{20}{c}} {{G_{{\mathrm{skin}}}} = \frac{1}{{{R_{{\mathrm{skin}}}}}}} \end{array}\end{equation*}

where ${V_{\mathrm{s}}}$ is the source voltage value, ${R_6}$ is the resistance of the resistor used in the voltage divider, and ${\mathrm{GS}}{{\mathrm{R}}_{{\mathrm{OUT}}}}$ is the measured voltage (supplementary figure S1). The validation and calibration of the GSR measurement was performed using various resistors, with known resistance values within the range of skin resistance (supplementary table S5), as known from physiological studies [20]. These values were chosen to characterize the behavior of the output voltage and its dependence on skin resistance. These measurements were used to produce a calibration curve for each glove to correct for bias and inaccuracy.

### 2.4. E-glove data sampling

To sample vital data, a clean e-glove was placed on each participant’s hand and secured with Velcro straps. The correct size of the fingertip holder for the HR sensor was determined for each participant to help obtain accurate readings, and the HR sensor was placed on the pointer finger. Two new electrode pads were placed on the participant’s middle and ring fingers and connected to the electrodes. A Velcro strap was used to secure the electrode pads to the fingers. Participants were instructed to remain still during data collection.

To begin data collection, the glove was switched on, and the OLED data display was used to confirm good signal from all sensors. The Bluetooth data collection script was run to begin recording data. Each participant had their vital data recorded for 15 min, while at rest. During the sampling, the TEWL was recorded 4 times for each participant by gently pressing the TEWL chamber against the skin of the hand for 30 s.

### 2.5. EBV collection and gas chromatography-mass spectrometry (GC-MS) analysis

Exhaled breath VOCs were collected and analyzed following our previously described protocol with minor modifications [4]. Participants avoided eating or drinking for at least one hour before sampling. Each participant exhaled tidally onto a 5 l Tedlar bag under the supervision of trained personnel. After that, 2 l of exhaled vapor was transferred into a Tenax TA thermal desorption (TD) tube using a calibrated VOC sampler (100 ml min^−1^ flow rate) [25]. Two tubes were collected per participant to generate technical replicates.

TD-GC-MS analysis was performed using a Gerstel TDU 2 TD unit coupled to a CIS 4 cryo-focusing inlet and MPS autosampler. Desorption started at 30 °C (3 min hold), ramped at 300 °C min^−1^ to 280 °C (5 min hold), and transferred volatiles through a heated line (280 °C) to the CIS held at −100 °C. Volatiles were then injected in a splitless mode onto the GC column as the CIS was heated at 12 °C s^−1^ to 280 °C (3 min hold). Chromatographic separation was achieved on an Agilent 6890N GC with a DB-5 ms column (30 m × 250 *μ*m × 0.25 *μ*m). The oven was programmed as follows: 38 °C (3 min), ramp 3 °C min^−1^ to 110 °C, 5 °C min^−1^ to 170 °C (1 min hold), then 20 °C min^−1^ to 280 °C (4.5 min hold), for a total runtime of 50 min. Helium was used as carrier gas at a constant flow of 1.8 ml min^−1^. Detection was performed on an Agilent 5975C single quadrupole MS operating in scan mode (m/z 30–350), with ion source and quadrupole temperatures of 230 °C and 150 °C, respectively.

Sample injections were randomized to minimize bias. Instrument performance was monitored by regular analysis of a Grob test mix and a *C*_7_–*C*_30_ alkane mixture to verify retention time stability and calculate Kovats indices. System blanks and blank TD tubes were analyzed every 10 and 20 runs, respectively, to confirm absence of artifacts.

### 2.6. Data preprocessing and modeling

#### 2.6.1. Vital sensors preprocessing and feature extraction

The sensors on the e-glove sample at a rate of 20 Hz. A moving-average window 1 s in length was used to smooth the signals, reducing sensor noise resulting from patient movement. No other post processing was done on the temperature and relative humidity data. Missing values from the heart rate data were interpolated from the surrounding datapoints.

Linear summary statistics were calculated from the heart rate and skin temperature data, such as minimum, maximum, mean, and standard deviation for a given patient. For the GSR data, a Python package called cvxEDA [26] was used to decompose the signal into the tonic and phasic components, and to estimate the sudomotor neuron activity (SMNA), which has been shown to directly correlate to sympathetic nervous system activity [20]. From the decomposed signal, various features were extracted, such as the number of phasic peaks, and the estimated number of SMNA peaks. An algorithm was developed for automatically detecting TEWL peaks in the skin chamber humidity data. The algorithm detects rapid increases in humidity to pinpoint the time of skin contact and calculates the change in humidity over time. Then, using a calibration curve, the ΔRH% is used to calculate the TEWL in each instance. Summary statistics, such as mean TEWL value, were then calculated for each patient. A full list of vital statistics can be found in supplementary table S7.

#### 2.6.2. EBV metabolomics, deconvolution, and data curation

Raw GC-MS data were deconvoluted, integrated, and aligned using an open-source MS-DIAL package (v4.9, RIKEN, Japan [27]). Tentative compound identifications were based on spectral matching against the NIST 2020 library and comparison of experimental Kovats retention indices. Peaks below 300 counts and siloxane-related base peaks were excluded prior to analysis. To eliminate background, compounds detected in blank samples (blank GC-MS runs and blank-clean sorbent tubes) were removed when its ratio sample/blank was lower than 10 times. Features that appeared in less than 25% of the samples were also removed to eliminate sparsely detected variables and enhance the reliability of subsequent multivariate analyses. A final peak table with peak intensities was created containing samples and features or variables in columns and rows, respectively.

#### 2.6.3. Vital sensors and EBV metabolomics MVA

Data processing and statistical analysis of the corresponding vital sensors and EBV metabolomic datasets were performed using MATLAB R2024b and PLS_Toolbox (version R9.0, Eigenvector Research Inc.). Principal component analysis (PCA) was first used to obtain an overview of the data, visualize group differences, and identify potential outliers. The vital data for one control sample was found to have a high Hotelling *T*^2^ score, but further inspection revealed that the abnormalities were not a result of device malfunction, so the datapoint was included.

Vital sensor data were *z*-score normalized, and peak intensities from EBV data were area normalized and log_10_-transformed, pareto-scaled and mean-centered prior to Partial Least-squares Discriminant Analysis (PLS-DA) analysis.

PLS-DA was applied as a supervised method to maximize separation between predefined sample groups. Model performance was evaluated using the area under the receiver operating characteristic curve (ROC AUC), along with model sensitivity and specificity. To assess model robustness, repeated random sub-sampling validation was performed (*n* = 50) in which samples were randomly divided into training (66%) and validation (33%) sets in each iteration.

Feature selection of potential biomarkers differentiating asthma and control groups was performed based on variable importance in projection (VIP) scores derived from the PLS-DA model. VIP values summarize the contribution of each feature to the model, with values greater than 1 used as a threshold to identify influential variables.

#### 2.6.4. Data fusion data processing

To perform the low-level data fusion, the EBV data were first area normalized and log_10_-transformed. The vital data from each patient were then concatenated to both replicates of their EBV data to produce a combined dataset, which was then *z*-score normalized (feature-wise) prior to repeated modeling with PLS-DA, as described above*. Z*-score normalization was used to prevent either dataset from dominating the results, due to differences in scale and variance. This simple concatenation of the two datasets is a form of low-level data fusion.

The mid-level data fusion was performed by generating features from the two data streams using PCA. The data were first pre-processed, as described above, before being fit with a PCA model. Due to small sample sizes, only 2 principal components were used as a reduced feature set for each data stream. This number of PCs was chosen to reduce the RMSECV of the PCA model. The 2 principal components of the EBV data captured 33.4% of the variance within the data, and the principal components of the vital data captured 50.1% of the variance within that data. These principal components were then concatenated to produce a combined dataset of 4 features, which were pareto-scaled and mean-centered before being used to build PLS-DA models with repeated test validation (*n* = 50) as with the other datasets.

## 3. Results

Table 1 summarizes the PLS-DA classification abilities obtained with the corresponding data. Permutation testing was used to show that the results were better than random chance, based on ROC AUC. The vital sensors statistics dataset included 39 samples and 20 variables. After VIP-based feature selection, 8 variables were retained for calculating the final classification performance. For the EBV metabolomics dataset, 78 samples (analyzed in duplicates) and 535 variables were initially evaluated. After variable reduction using VIP scores, 135 variables were selected for building the final PLS-DA models. Supplementary table S8 gives the retention indices of these VOCs, some of which have been tentatively identified.

**Table 1.** Results of analysis of individual data streams and the combined datasets.

Dataset	Reduced feature set size	Sensitivity	Specificity	ROC AUC
EBV metabolomics	135	0.68 (0.14)	0.63 (0.15)	0.72 (0.10)
Vital sensors	8	0.62 (0.20)	0.53 (0.18)	0.59 (0.12)
Combined data (Low-level fusion)	135	0.80 (0.13)	0.82 (0.14)	0.88 (0.07)
Combined data (Mid-level fusion)	4	0.61 (0.16)	0.44 (0.17)	0.59 (0.10)

The low-level data fusion for the vital sensors and EBV metabolomics data was performed by concatenation of feature arrays. 18 and 117 features were retained from the vital sensors and EBV data, respectively, in the combined dataset following VIP feature selection, producing an augmented array of 135 features. The most important vital statistics included following VIP feature selection were those related to the GSR signal, such as the estimated number of GSR SMNA peaks.

As expected, the exhaled breath metabolomics provided some predictive power for identification of asthma. The models trained on EBV data performed better than random chance, as compared to the uninformed model obtained using permutation testing (*p* < 0.001). The vital data had less predictive power than the EBV data in classifying asthma, based on ROC AUC (*p* < 0.001), using Mann-Whitney U test, but still performed better than random chance (*p* = 0.007). The mean ROC AUC of the low-level fusion models was significantly higher than the EBV-only model (*p* < 0.001). The mid-level data fusion models also performed better than chance (*p* < 0.001) but did not have significantly different results than the vital-only model (*p* = 0.72). Figure 3 summarizes boxplots of the predicted PLS-DA *Y*-values for each of the models, where controls (*Y* = 0) and asthmatics (*Y* = 1) are compared to a threshold value (TH). Additionally, figure 4 shows the ROC curves of the models studied. In this case, we can observe the differences by sensitivity (true positive rate) and 1-specificity (false positive rate) for each dataset.

**Figure 3.**
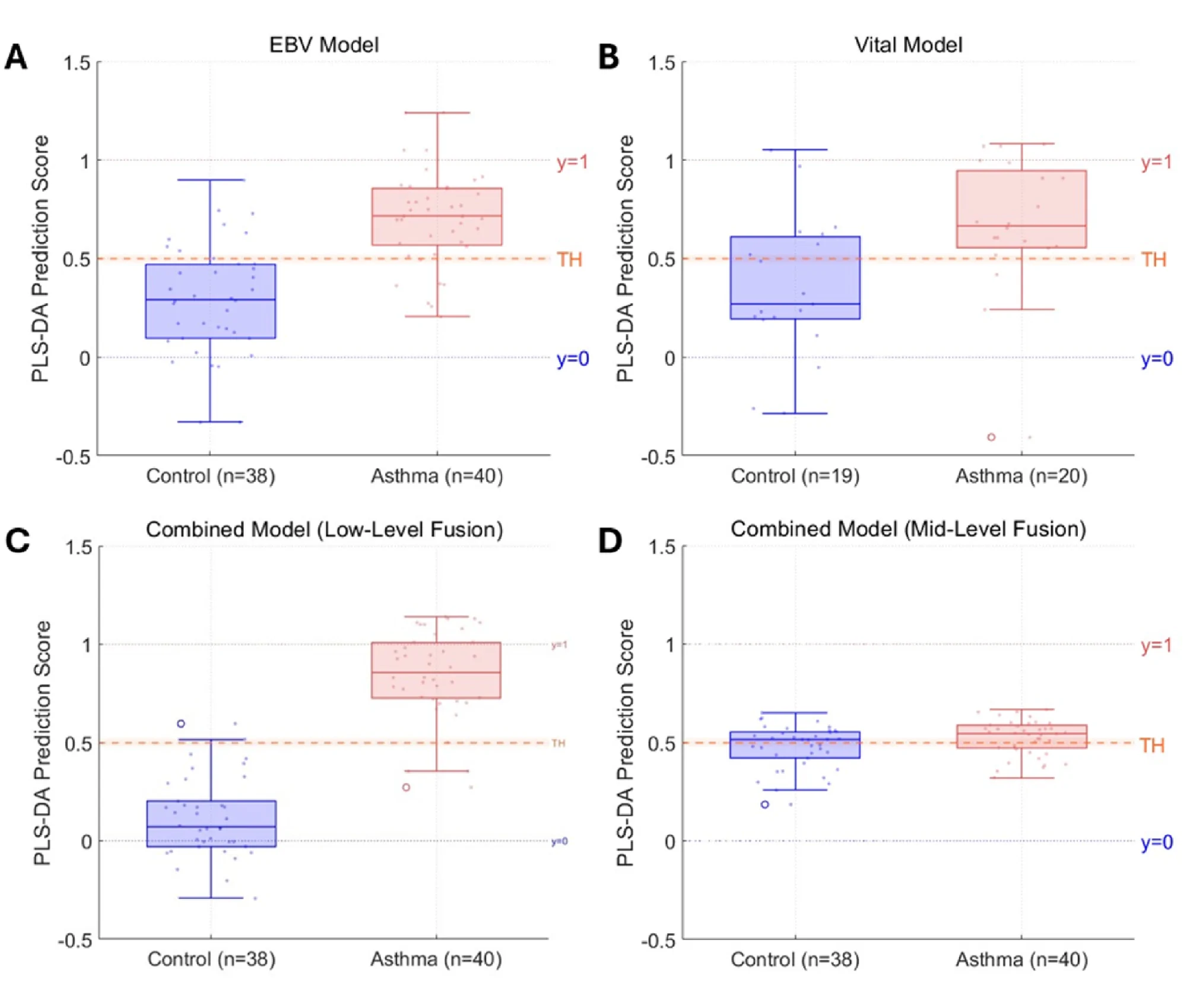
Box and whisker plots of the PLS-DA predictions scores obtained from the (A) EBV data, (B) vitals, (C) the low-level fusion combined dataset, and (D) the mid-level fusion combined dataset. Models including EBV have twice as many samples as breath was measured in duplicate whereas e-glove measurements were collected once. Also note that scores shown include calibration and validation results.

**Figure 4.**
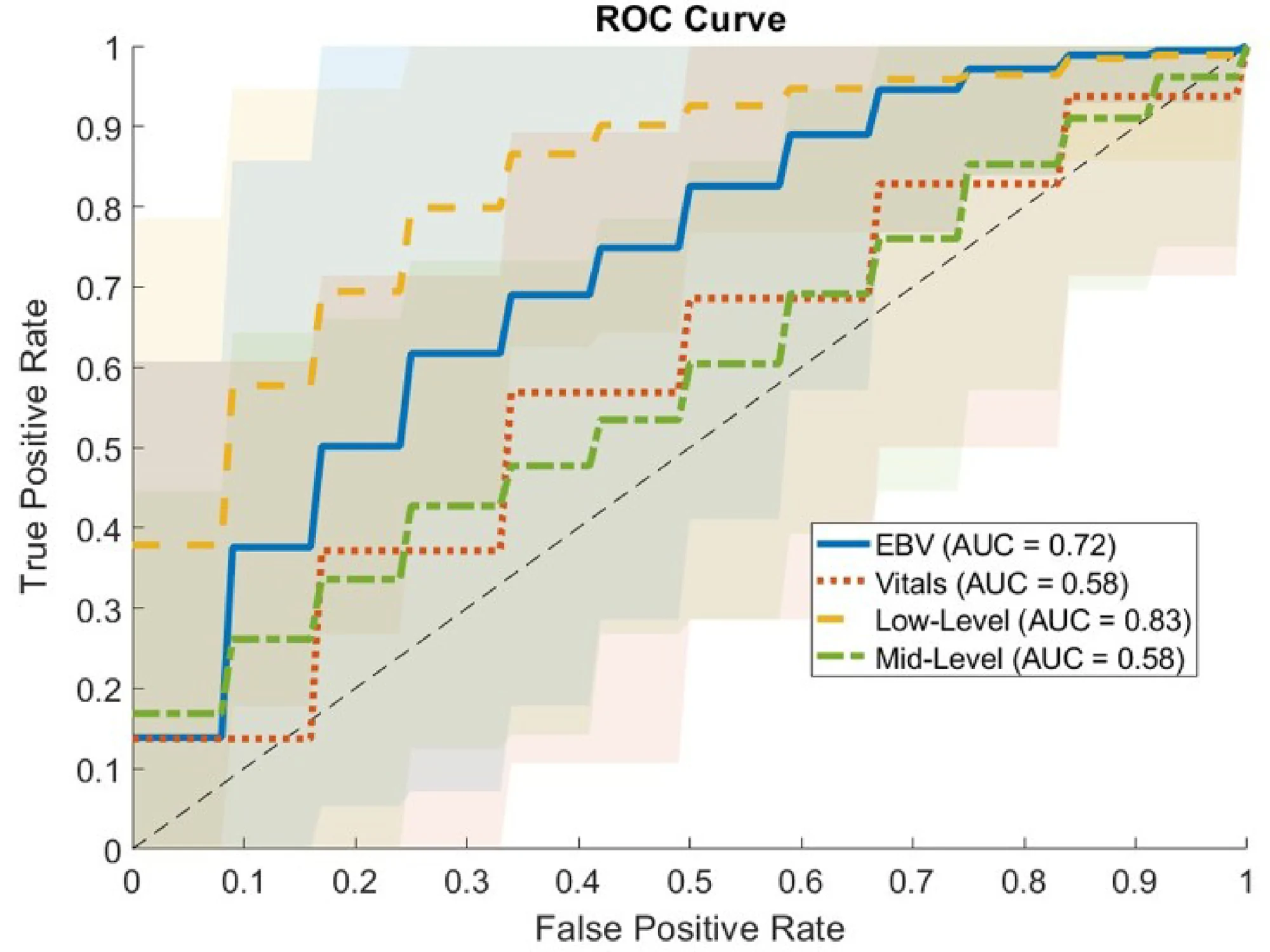
Representative receiver operating characteristic curves for each dataset. Curves are 50 repeat averages with 95% confidence bands. AUC scores are for the particular ROC curve shown.

## 4. Discussion

The results of the data fusion show that the low-level fusion model presented significant improvement over the EBV-only model, based on ROC AUC. This improvement in the low-level fusion model suggests that it was able to capture more information about asthma in the participants studied. Low-level fusion also significantly outperformed the mid-level fusion approach, in this case. This result suggests that low-level data fusion is a strong option for small, high-dimensionality datasets, where other data fusion approaches requiring supervised, cross validated models, are not practical due to dataset size limitations. However, the improvement in the fused model was relatively modest. It is possible the information contained in the vital statistics is already mostly captured by the metabolomics data. A larger cohort of both controls and asthmatics may improve the power of the models as well as the statistical power of tests used to determine their accuracy.

The vital features retained in the low-level fusion dataset following VIP feature selection were calculated from the heart rate, body temperature, and GSR signals. The features calculated from the decomposition of the GSR signal were particularly influential in the model. Further exploration of these signals, perhaps by engineering new features to capture more information, might lead to improved performance. For example, recent work has shown the applicability of nonlinear analysis of vital signals for the diagnosis of disease. Nonlinear features, calculated from time-series data, such as largest Lyapunov exponent, can provide insight into the dynamics of a complex system like the human body [19]. Additionally, the inclusion of other vital features derived from measures more closely related to asthma severity, such as breathing rate or airway noise, may improve the results. This is a strong avenue for future research, as improving the information content of the vital statistics is key to more accurate health estimation data fusion work.

The low AUC of the PLS-DA models produced by the mid-level fusion suggests that the mid-level fusion dataset contained less information than the other datasets. PCA is useful for mid-level fusion of small datasets because it is unsupervised and does not require a separate calibration set for transforming the data. However, in this case, it appears that the principal components selected failed to capture the differences between controls and asthma patients. It is also possible that the dimensionality reduction inherent in PCA too strongly regularized the data and made it ineffective for classification purposes. Exploration of other data fusion strategies is a likely avenue of pursuit. In future work, larger datasets may also allow for more complex, multi-stage analyses with more data to use for cross-validation and test sets. Larger datasets will also allow for the use of more advanced classification models with lower chance of over-fitting on the calibration set.

Age presents a major confound in this study, with the average age in our asthma cohort being 18.5 years older than our control cohort. Physiological variables, like those in our vital statistics, are known to change with age [28]. Additionally, VOC profiles in breath are also likely affected by patient age [29], meaning that the overall results should be interpreted with caution. It is also unclear to what extent other confounding effects or co-morbidities in the asthma cohort may have affected the results.

Future work might investigate the effect confounding factors can have on patient biomarkers. As more data is collected, the impact of co-morbidities, age, and medication use on patient vital statistics and EBV metabolite profiles may become clearer and can potentially be accounted for to improve classification capabilities. Asthma severity is also known to vary throughout the day, and from one day to the next. Ensuring consistent time of day in breath collection may reduce variability between patients, and taking standard biomarkers, such as a FeNO measurement, at the time of breath collection may help account for variations in severity [30]. Diet can also be a confounding factor in breath analysis. Our work uses a comparatively short 1 h fasting window prior to collection. Accounting for diet, skincare, and grooming products may improve classification accuracy. Attempting to account for inhaled volatiles from room air may also improve results [31, 32]. Additionally, larger patient cohorts will enable further subdivision by asthma phenotype, potentially elucidating differences in VOC profiles by subtype.

Overall, each dataset produced models with higher sensitivity than specificity. This indicates that the models had more difficulty classifying healthy controls as compared to asthma patients. There is an inherent trade-off in sensitivity and specificity. Future work should consider the potential cost of false positives and false negatives in screening for and monitoring the conditions of asthma patients. Given the relatively low-risk nature of asthma diagnosis and intervention, trading higher sensitivity for lower specificity may be appropriate for these models.

This work highlights the potential of data fusion techniques in the development of new health monitoring methods. As new sensing modalities and data analysis techniques emerge, exploring ways to maximize their efficacy will be key in facilitating their adoption into clinical practice. Data fusion is an important component of this exploration, and more work is needed to fully realize its promise.

## Data Availability

All data that support the findings of this study are included within the article (and any supplementary files). Supplementary eGlove Data available at https://doi.org/10.1088/1752-7163/aeb685/data1.
